# Medical Proceedings in the State of Connecticut*Proceedings of the President and Fellows of the Connecticut Medical Society, in Convention, with a list of the members, for the years 1834, 35, 36, and 37.Report of the New Haven County Medical Society, on the expediency of repealing that section of the medical laws of the State, which excludes irregular practitioners from the benefits of law in the collection of fees.

**Published:** 1837

**Authors:** 


### Art. II.—Medical Proceedings in the State of Connecticut.*

We are indebted to Dr. Charles Hooker, Secretary, for these pamphlets. They present the medical gentlemen of the ancient and orderly state of Connecticut, as organized by legal enactments into a corporate body, which, within the last year, has been exerting itself (we understand successfully) to prevent a relaxation in the laws prohibiting quackery. In his accompanying letter, Dr. Hooker asks, why we do not unite in a similar way in Ohio? The answer is, that for twenty years from 1810 or '11, there was a law regulating the practice of physic in our state, and that it was repealed from a conviction on the part of our legislature, generally concurred in by the profession, that it was unproductive of good. The people are fond of quackery, and in every instance, as far as we know, in which an attempt was made to enforce its penalties, they took sides with the empyric. The societies, district and state, which it created, were succeeded by the *voluntary* Medical Convention, which met at Columbus in January, 1835, and is to meet again on the first Monday of January next. The important proceedings of that convention were made known to the public by this journal. We trust that the next meeting will be attended by a still larger number

---

* Proceedings of the President and Fellows of the Connecticut Medical Society, in Convention, with a list of the members, for the years 1834, 35, 36, and 37.

Report of the New Haven County Medical Society, on the expediency of repealing that section of the medical laws of the State, which excludes irregular practitioners from the benefits of law in the collection of fees.

than the first, and that what was so auspiciously begun, will be carried on with dignity, spirit and liberality. But let us return to the proceedings of our Connecticut brethren. Appended to those of 1837, is an address to the Annual Convention, by the very intelligent and respectable Dr. Miner, its president. Its drift is the dignity of the profession. On the subject of united effort, he holds the following just and forcible language:

"Nothing great and extensively good can ever be done in the world, without association, combination, and united effort. To this end, if the good is to be permanent, the associations must have a two-fold bearing; they must be both beneficial to the public, and to themselves. If they are not beneficial to the public, in free governments the public will not long tolerate them; and if they are not beneficial to the associations, the members will not long be at the trouble and expense of meeting. There is not, therefore, the slightest clashing between the public and all proper organized societies. Indeed, it is impossible to promote their mutual interest in any other way than by such organizations. These associations form one of the most prominent distinctions between civilization and barbarism, and no extensive improvement can be diffused without such institutions."

On the *right* of legislatures to prohibit quackery, Dr. M. thus expresses himself.

"Legislators have certainly the right to interfere, whenever the public good is at stake; and it is their main business to protect individuals, where individuals have not the power to protect themselves. They protect from fraud and imposition, as well as from other injuries, by placing barriers against dishonesty. Thus, they either do, or may, direct the inspection of every kind of provision, lumber or other article of merchandise, before it can be lawfully exposed in market. They direct the examination of all officers of the army and navy, previous to their receiving commissions. In most countries, they examine the qualifications of gentlemen of the bar, to prevent clients from being imposed on by pettifoggers. They commonly insist upon the examination of teachers, before they can become lawful instructors. In most parts of the civilized world, even a common trade cannnot be exercised, unless the master has complied with certain forms, which amount to a strict examination.

"All these regulations are designed for the benefit of the people in general, and not directly for the benefit of the trades or professions. It is supposed that individuals could not have time, if they possessed sufficient knowledge, to examine the quality of every barrel of flour, beef, pork and fish that they buy, before purchasing them. Nor is it wished to have the client lose his suit, to discover that he has employed an incompetent advocate; or for schooling to be found to be worse than useless, from having employed an incompetent teacher.

"I can see no reason, why the health of the community, and consequently public protection against ignorant and unprincipled pretenders—for pure quacks, whatever good qualities they may sometimes possess, from the very nature of the case must always be knaves, since they only flourish by the falsest pretensions and most barefaced impositions—are not a subject of legal regulation, as much as the army, navy, schools, trades, manufactures, and in a word every other matter, in which individuals are either unable or indisposed to protect themselves.

"Besides, no power on earth can give a quack or other impostor a *moral right* to his fraudulent gains; should the state, therefore, from mistaken policy, legalize his claims, it would amount to granting a bounty to dishonesty, and as far as it goes, would serve to sap the principles of morality. It is impossible to change the moral aspect of empiricism, or of any other fraud, whatever may be the supposed policy, as to legislative interference. A quack cannot be converted into an honest man, by any law whatever, much less can he be made learned against his will."

We hold it impossible to gainsay these observations, and wish they could be carried home to every legislator, and, what would be more important, to every constituent. But, to reach the people, physicians must simultaneously exert their influence. Hear what Dr. M. says on this subject:—

"One part of the duty of professional men, and that by no means the least, is to instruct and inform the public mind. Professional men are a kind of missionaries in the civilized world. In the division of labor, not for their own benefit, but for the public good, they are the great teachers of mankind. It is the practice of all legislative bodies occasionally to consult them, and it is just as necessary to obtain the opinions of physicians before making laws for preserving health, as it is to advise with merchants, manufacturers and mechanics, concerning their several departments. It devolves upon our profession to teach the public the nature of quackery, and to expose the absurdities and dishonesty of empiricism. This duty is now peculiarly incumbent upon *us*."

The Report of the New Haven County Society is principally directed against the repeal of the law which is designed to embarrass, if it cannot suppress quackery, by refusing legal process to quacks for the collection of their *professional* fees. As a specimen of the manner in which the subject is treated, we give this extract:—

"The reason that imposition is so easy and successful in medicine, is to be found in the general want of information regarding the nature of disease, the operation of remedies, and the powers of the human system. As a consequence, the skill and knowledge of a physician must, for the most part, be taken upon trust, except so far as evidence is to be obtained from his general character and acquirements. There is no subject which, by the mass of mankind, is so rarely made a matter of general study and investigation, even in its elements, as medical science; while, at the same time, there is no subject regarding which men so universally consider themselves adequately informed, in all its practical applications, as this same medical science. This want of knowledge, unfortunately not felt as a *want*, we deplore, because worth in our profession is, as a consequence, imperfectly appreciated, and because ignorance and impudence thereby gain an advantage of the utmost importance. It is well known, that the arts of intrigue, and the no less potent art of puffing, will oftentimes procure occupation and a name, when unpretending merit is left to perish unnoticed. This fact is well illustrated by the sudden and full employment frequently obtained by itinerants and adventurers without character or merit, and of whose vaunted skill and cures we know nothing, except what is to be found in a pompous advertisement or handbill."

The latter part of the Report is directed upon *Samuel Thompson*. It appears that one of his agents, in the West, in three and a half years sold patent rights to the amount of $80,000 !! A most liberal reward by the good people, for *permission* to inflame their stomachs with cayenne pepper, cloves, ginger, and alcohol. By the way, there can be but little doubt that the stimulus which Thompson recommended, was the great secret of his popularity. As our temperance societies rendered drinking unpopular and disreputable, a course of medicinal stimulants came in very opportunely. "No. 6" indeed, took the place of our favorite old whiskey, and men and women excited themselves by the advice of the general government! All hail, the march of mind! D.

